# Association Between Adverse Childhood Experiences, Intimate Partner Violence, and Nutritional Inadequacy Among Women in an Urban Brazilian Setting

**DOI:** 10.3390/ijerph22091413

**Published:** 2025-09-10

**Authors:** Nathália Miguel Teixeira Santana, Franciéle Marabotti Costa Leite

**Affiliations:** Department of Public Health, Lavisa, Federal University of Espírito Santo, Vitória 29043-900, Brazil; nathalia.miguel@hotmail.com

**Keywords:** body mass index, intimate partner violence, adverse childhood experiences

## Abstract

Adult women’s nutritional status reflects a complex interplay of biological, social, and environmental determinants. This study aimed to analyze the association between adverse childhood experiences (ACEs), intimate partner violence (IPV), and inadequate nutritional status among women in Vitória, Espírito Santo, Brazil. We conducted a cross-sectional, population-based survey with 1.073 women aged 18 years or older. The body mass index (BMI) is categorized into adequate weight and inadequate weight, with the latter comprising excess weight (overweight and obesity) and extremes of weight (underweight or obesity). Experience of violence was measured with ACEs and IPV. Logistic regression analyses were performed to identify associations, adjusting for sociodemographic variables. Women who reported ACEs, such as parental divorce/separation and living with family members who used alcohol, illicit drugs, or prescription drugs, were 1.4 times more likely to be excess weight compared with those who did not experience ACEs (OR = 1.37; 95%CI:1.02–1.83; *p* = 0.035 and OR = 1.35; 95%CI:1.01–1.80; *p* = 0.041, respectively). Among those with extreme BMI values, the association with parental divorce/separation also remained significant after adjustment (OR = 1.59; 95%CI: 1.13–2.25; *p* = 0.009). These findings suggest that ACEs have lasting effects throughout life, influencing body weight in adult women and contributing to inadequate dietary status at both the lower and upper limits.

## 1. Introduction

Violence is a complex public health problem with significant short-term, medium-term, and long-term effects [1]. In childhood, exposure to adverse experiences can impair healthy development and lead to physical, cognitive, emotional, and social harm [2]. These childhood adversities can have lifelong repercussions, extending into adulthood and impacting both physical and mental health [3]. Among adult women, intimate partner violence (IPV) represents a particularly prevalent form of violence, which also has significant health consequences [4].

Globally, approximately one-third of adult women have experienced at least one episode of physical and/or sexual violence, whether perpetrated by intimate partners or others [5]. In low- and middle-income countries, the prevalence of IPV is estimated at 37.2% [6]. In Brazil, data from the 2019 National Health Survey reported a 7.6% prevalence of IPV among women aged 18 to 59, with marked regional disparities [7]. In the city of Vitória, Espírito Santo, the prevalence of IPV throughout life and in the past year in 2014 was 57.6% and 25.3% for psychological violence, 39.3% and 9.9% for physical violence, and 18% and 5.7% for sexual violence, respectively [8,9].

In recent years, studies have increasingly explored the association between violence and health outcomes, including its relationship with food insecurity [10] and nutritional status, particularly obesity [11], a condition that has reached epidemic proportions worldwide. Evidence suggests that people exposed to adverse childhood experiences (ACEs) are at higher risk of developing obesity in adulthood, with a more significant impact among women compared to men [12]. Women who experienced four or more adverse events had a 44% higher risk of developing obesity in adulthood compared to those who did not experience any events [13]. However, gaps remain regarding the identification of the specific types of adversity that are most strongly associated with nutritional outcomes. Most studies focus on the cumulative number of adverse events over the life course, often overlooking the distinct nature of individual experiences [14].

Beyond early-life adversity, IPV has also been linked to both undernutrition and overweight, particularly among women. In India, women exposed to high rates of physical and sexual IPV were 24% more likely to be malnourished [15]. Similarly, in Ethiopia, women who had experienced sexual IPV were almost three times more likely to be underweight compared to those who had not experienced this form of violence [16]. In Brazil, it was observed that women who had experienced physical IPV had lower body mass index (BMI) values compared to those without this experience [17].

In Nepal, victims of sexual IPV were 2.6 times more likely to be underweight, while women exposed to physical IPV were at higher risk of overweight/obesity [18]. In Bangladesh, women with inadequate nutritional status showed positive associations with different forms of IPV [19]. On the other hand, a study in Zimbabwe showed that women who experienced at least one form of IPV (physical, psychological, or sexual) were more likely to be obese [20].

Despite its complexity, violence at any stage of life is preventable. Understanding how childhood adversity and IPV influence nutritional outcomes in adulthood can help identify high-risk groups and highlight new approaches to prevention and treatment.

Therefore, this study aims to investigate the association between ACEs, lifetime exposure to different forms of IPV, and inadequate nutritional status among adult women residing in Vitória, Espírito Santo, Brazil.

## 2. Materials and Methods

This is a cross-sectional, analytical, population-based study carried out in the city of Vitória, Espírito Santo, Brazil. The municipality of Vitória, the capital of the state of Espírito Santo, had an estimated population of 322,869 people as of 2022. According to data from the 2022 census, the female population accounted for 53.71% of the total residents, comprising 173,415 women [21].

The study population consisted of women selected through multistage cluster sampling based on census tracts. Households characterized as exclusive and mixed, with at least one female resident, were eligible for the study. A random draw was conducted to select households with more than one resident for the interview. Information of interest was collected through face-to-face interviews with the selected woman in each household. All interviews were conducted in a private setting within the household, without the partner’s presence, and were conducted exclusively by female interviewers, in accordance with World Health Organization (WHO) recommendations.

The pilot study took place in December 2021. The data collected in the pilot study were not part of the final research sample. Fieldwork began after the data obtained in the pilot study were analyzed.

Women aged 18 and older who had had an intimate partner within the last 24 months prior to the interview were eligible. Intimate partners were current or former partners and/or boyfriends if they were having sexual relations, regardless of formal legal union. Those unable to understand or communicate due to intellectual or sensory deficits, and therefore unable to respond to the instrument, were excluded. Fieldwork took place between January and May 2022, involving 1086 women. For this study, three participants were excluded due to incomplete weight and/or height data, and another ten due to BMI values that were more than three standard deviations above the mean. No covariates included in the analysis contained missing data, resulting in a final sample of 1073 women.

The dependent variables were derived from self-reported anthropometric data collected during face-to-face interviews. Participants were asked to report their current weight in kilograms (kg) and height in meters (m). The BMI was calculated as weight divided by height squared (kg/m^2^), following the criteria established by the WHO.

For analysis, nutritional status was classified and organized into specific groups:

Adequate weight: BMI between 18.5 and 24.9 kg/m^2^ (normal weight).

Excess weight: BMI ≥ 25 kg/m^2^ (overweight (BMI between 25 and 29.9 kg/m^2^) and obesity (BMI ≥ 30 kg/m^2^)).

Extreme BMI: BMI < 18.5 kg/m^2^ (underweight) or ≥ 30 kg/m^2^ (obesity).

Women with a BMI within the normal weight range were classified as having adequate nutritional status. At the same time, those falling into either of the two other categories were considered to have inadequate nutritional status.

ACEs were assessed using a subset of the WHO Adverse Childhood Experiences International Questionnaire (ACE-IQ), which has been translated, adapted, and validated for use among Brazilian adults [22]. The questionnaire included items related to the participant’s childhood and adolescence (up to 18 years of age), covering key categories and subcategories of adversity, such as parental relationship (physical and emotional neglect); abuse (emotional, physical, and sexual); and family environment (alcohol, illicit drug, or controlled medication use; depression, mental illness, or suicidal ideation; incarceration of a family member; parental separation or divorce; and death of parents or guardians). Responses were measured on a frequency scale: never, rarely, sometimes, often, or always. These responses were later dichotomized as “no” for “never” and “yes” for all other options. An adverse childhood experience was considered present if the respondent reported at least one affirmative event within a given subcategory.

To evaluate lifetime exposure to IPV throughout life, the study used the WHO Violence Against Women (WHO VAW STUDY) instrument, which has been translated and validated in Brazil [23]. The questionnaire consisted of 13 items: 4 related to psychological violence, 6 to physical violence, and 3 to sexual violence. All items had binary response options (yes/no). A woman was considered to have experienced IPV if she responded “yes” to at least one item in any of the three categories.

Sociodemographic variables were included in both continuous and categorical forms: age in completed years (≤29; 30–39; 40–49; 50–59; ≥60); years of education (0–8; 9–11; ≥12 years); family income in Brazilian reais (categorized into tertiles, with the first representing the lowest income and the third the highest); and race/skin color as a categorical variable (white or non-white).

The study was approved by the Research Ethics Committee on Human Subjects at the Federal University of Espirito Santo (approval number 4,974,080, September 2021). All participants were informed about the study procedures and signed a written informed consent form, which ensured anonymity, confidentiality, and the right to refuse or withdraw from participation at any time.

A descriptive study was performed, presenting absolute and relative frequencies. Bivariate analyses were performed using the chi-square test and Student’s *t*-test. Multicollinearity was assessed using the variance inflation factor (VIF), with a threshold of VIF < 5 considered acceptable. Logistic regression models were conducted to estimate the association between ACEs, IPV, and inadequate nutritional status. Crude and adjusted odds ratios (ORs) and 95% confidence intervals (95% CIs) were calculated. The variables associated with the outcomes under study were entered at levels according to the bivariate analysis (*p* < 0.20). The adjusted analysis was conducted by entering the variables into the model at three levels to account for possible confounding factors. Sociodemographic variables (age, race/color, education, family income) were initially included in the first models as potential confounders. In the second, the data were used to build the sequential models, beginning with the inclusion of sociodemographic covariates, followed by individual ACEs (e.g., parental incarceration, parental separation or divorce, substance abuse in the household, and family member with depression or mental illness). In the third step, the data of IPV variables (physical and psychological) were then added to the final adjusted model. The final model adopted a significance level of 5% (*p* < 0.05). All analyses were performed using Stata^®^ version 14.0 (StataCorp, College Station, TX, USA).

## 3. Results

The final sample included 592 women (56.9%) with excess weight and 449 women (43.1%) with normal weight. Regarding extreme BMI categories, out of 268 women (37.4%), 32 (4.5%) were underweight, and 236 (32.9%) had obesity.

Table 1 presents the characteristics of women categorized by BMI. On average, participants with excess weight were older and had higher body weight, but lower height, family income, and education levels (*p* < 0.05) compared to those with normal weight. A higher proportion of non-white women was also observed in the excess weight group (*p* = 0.009). Regarding childhood adversities, a greater frequency of alcohol, illicit drug, or controlled medication use in the family environment was reported (*p* = 0.002).

Women classified as having extreme BMI had lower height, family income, and education levels (*p* < 0.05), along with higher body weight and a higher proportion of non-white individuals. Compared to women with normal weight, they reported a higher prevalence of childhood adversities, including physical neglect, family history of substance abuse (alcohol, illicit drugs, or controlled medications), incarceration of a family member, and parental divorce or separation (*p* < 0.05). The prevalence of psychological (*p* = 0.048) and physical (*p* = 0.007) IPV was also higher among women with extreme BMI compared to those with adequate BMI.

Table 2 describes the distribution of women with excess weight (BMI ≥ 25 kg/m^2^) and extreme BMI (BMI <18.5 or ≥30 kg/m^2^) according to sociodemographic factors, childhood adversities, and lifetime IPV. Women with excess weight were more prevalent among older age groups (50–59 years), those with lower education (0–8 years), and those in the second tertile of income levels (*p* < 0.05). A similar pattern was observed for women with extreme BMI, except for household income, which is more prevalent among those in the first tertile (*p* < 0.05).

Regarding childhood adversities, a significantly higher prevalence of excess weight and extreme BMI was found among women reporting a history of alcohol, drug, or controlled medication use in the family environment (*p* = 0.002 and *p* = 0.004, respectively). Extreme BMI was also associated with reports of physical neglect (*p* = 0.036) and incarceration of a family member (*p* = 0.038); however, the confidence intervals overlap, indicating an association only with parental separation or divorce (*p* = 0.003).

Regarding lifetime VPI with extreme BMI, psychological violence (*p* = 0.048) and physical violence (*p* = 0.007) were statistically significant; however, they do not indicate an association due to overlapping confidence intervals. No significant associations were found between other forms of lifetime IPV and inadequate nutritional status.

No evidence of multicollinearity was observed among the independent variables included in the regression models (all VIFs < 2.0). The crude and adjusted analyses for women with excess weight are presented in Table 3 (pseudo-R^2^ = 0.054). After controlling for potential confounders, women aged 40–49 and 50–59 years were approximately 2.5 times more likely to have excess weight compared to those aged 18–29 years. Lower educational attainment (0–8 years of schooling) was associated with nearly double the odds of excess weight compared to women with 12 or more years of education (OR = 1.88; 95% CI:1.24–2.84; *p* = 0.008). Belonging to the second income tertile was also associated with a higher likelihood of being overweight or obese compared to the highest tertile (OR = 1.80; *p* = 0.002). The association with race or skin color lost statistical significance after adjustment for other factors.

Regarding experienced childhood adversities, a history of alcohol abuse, illicit drug use, or use of controlled medications in the household remained significantly associated with excess weight (adjusted OR = 1.37; 95%CI:1.02–1.83; *p* = 0.035). Similarly, parental separation or divorce was linked to a higher likelihood in the adjusted model (OR = 1.35; 95% CI:1.01–1.80; *p* = 0.041). Exposure to lifetime IPV, including psychological, physical, or sexual violence, was not significantly associated with excess weight.

Table 4 presents the crude and adjusted analysis of extreme BMI. The model had a reasonable explanatory power (pseudo-R^2^ = 0.072) based on the included variables. The likelihood of having an extreme BMI increased with age and declined among women aged 50–59 years (30–39: OR = 1.74; 95% CI: 1.06–2.86; 40–49: OR = 2.17; 95% CI: 1.30–3.60, and 50–59: OR = 1.97; 95% CI: 1.15–3.37). We observed that, regarding education and monthly family income, the lower the level, the greater the chances of being classified as a woman with underweight or obesity (0–8 years: OR = 2.36; 95% CI: 1.42–3.92; *p* = 0.004; 1st tertile: OR = 2.29; 95% CI: 1.44–3.64; *p* < 0.001).

Among the adversities experienced in childhood, parental separation or divorce was associated with an increased likelihood of being at an extreme of the BMI in the adjusted analysis (OR = 1.59; 95% CI:1.13–2.25; *p* = 0.009). Other adversities, such as physical neglect, alcohol, illicit drug abuse, or controlled medication, and a history of family imprisonment, as well as psychological and physical IPV throughout life, did not maintain sigificance after adjustments.

## 4. Discussion

This study identified an association between childhood adversity, such as alcohol abuse, illicit drug use, or the use of controlled medication by family members, as well as parental separation or divorce, and inadequate nutritional status among adult women residing in Vitória, Espírito Santo.

Significant associations were observed between inadequate BMI and sociodemographic factors, including age, education, and family income. Women aged 40–59 years were found to be more than twice as likely to present with excess weight compared to those aged 18–29 years. These findings are consistent with previous studies addressing this issue among female populations [24,25]. However, it should be noted that weight gain tends to increase until a certain age, after which a decline is observed, as underweight status becomes more prevalent among older Brazilian adults [25].

Similar associations between educational attainment and nutritional status have been reported in prior research. Our findings corroborate research indicating an inverse relationship between educational level and the probability of excess weight [26]. Furthermore, women in the lowest tertiles were more likely to have inadequate BMI than those in the highest tertile, suggesting that even in low-income communities, economic differences may influence the risk of being overweight [24]. These findings support existing research that emphasizes the strong impact of social class and education level on vulnerability to IPV [27] and its subsequent effect on health outcomes.

ACEs and their impacts on life have been widely documented. The findings of our study are consistent with a cohort study that demonstrates an increased risk of obesity in adult women who experienced four or more ACEs, independent of other factors [13]. A meta-analysis also reported a heightened risk of adult obesity associated with childhood adversities such as physical abuse (OR: 1.60), sexual abuse (OR: 1.58), and psychological or non-physical abuse (OR: 1.31), with a more pronounced effect observed in women than in men [28]. These results support a dose–response relationship, whereby greater exposure to adverse events correlates with a higher risk of obesity and other adverse health outcomes in adulthood [28,29].

A study conducted among Norwegian adults found that individuals who experienced parental separation or divorce during childhood had an increased risk of reporting multiple childhood adversities [30]. This supports the hypothesis that parental separation may not only constitute an adverse experience itself but also increase the likelihood of additional adversities, thereby influencing adult nutritional status. Furthermore, research in pediatric populations revealed that children with obesity were significantly more likely to report multiple ACEs, including food or housing insecurity (OR: 1.64; 95%CI: 1.26–2.13), parental divorce (OR: 1.67; 95%CI: 1.32–2.13), living with family members with substance use issues (OR: 1.65; 95%CI: 1.24–2.20), and exposure to four or more ACEs compared to their peers (*p* < 0.001) [31]. These findings support the hypothesis that obesity may originate in childhood and persist into adulthood, influenced by the accumulation of early-life adversities. Recent reviews emphasize that psychobiological mechanisms may also explain this association. Dysregulation of the hypothalamic–pituitary–adrenal (HPA) axis has been identified as a key pathway that may mediate the relationship between childhood adversity and elevated BMI in adulthood [32,33].

Although this study did not find a statistically significant link between lifetime IPV and nutritional status, earlier research from various countries has increasingly shown a connection between experiencing violence—whether psychological, physical, or sexual—and its effects on body weight, including both underweight and overweight or obesity [15,16,17,18,19,20]. The lack of a significant association in the adjusted models shows there is no direct relationship between lifetime IPV and BMI in this sample; however, this trend deserves further exploration. It is possible that unmeasured mediating factors, such as violence-related stress [34] and the onset of depressive disorders [35], could impact eating behaviors and thus influence body weight [36].

The mechanisms associated with violence and nutritional problems are diverse and complex. Biologically, persistent stress from violence leads to elevated cortisol levels and other hormonal shifts that can affect metabolism and appetite [32,33]. Behaviorally, IPV may limit women’s control over their food decisions and increase food insecurity risks [20]. Moreover, psychosocial issues such as anxiety, depression, and post-traumatic stress disorder can directly or indirectly influence body weight regulation and eating behaviors [34,35,36].

It is important to highlight that in Brazil, several social assistance programs are crucial in tackling nutritional issues. The Bolsa Familia program plays a key role in food security by providing conditional cash transfers to low-income families. The Food and Nutrition Surveillance System (SISVAN) tracks the nutritional status of the population, enabling targeted interventions for vulnerable groups. Additionally, the National Health Promotion Program (PNPS) promotes healthy eating habits and the prevention of non-communicable chronic diseases, which helps improve nutritional outcomes and decrease health disparities.

Among the strengths of this study is its population-based design, which ensures representativeness of women residing in Vitória, Espírito Santo. Additionally, it addresses a relatively underexplored area—the intersection between different types of violence and BMI. Nonetheless, some limitations should be acknowledged. Anthropometric measurements were self-reported, which may introduce information bias, despite being collected through face-to-face interviews. Including both underweight and obesity in one category is also a limitation since these conditions differ in their causes and health impacts. The cross-sectional nature of the study also limits causal inference, particularly regarding IPV experienced during adulthood, which may have occurred after the onset of obesity, thus complicating the temporal interpretation of exposure and outcome. Furthermore, childhood adversities may present a memory bias and were not categorized by the number or accumulation of events, which limited comparability with other studies employing cumulative ACEs scores. However, this approach was chosen based on the understanding that the type of adversity experienced may exert distinct effects, and that aggregating such experiences may obscure significant differences. It is also important to note that since the study took place in an urban environment, the findings might not apply to women in rural areas or different parts of Brazil.

## 5. Conclusions

We conclude that childhood adversities are strongly linked to inadequate BMI categories—including underweight, overweight, and obesity—in adult women, emphasizing the important role of ACEs as key factors influencing long-term health outcomes. Our results highlight the need for care approaches focused on childhood trauma. Incorporating preventive actions along with nutritional and psychosocial support may help reduce the enduring impact of childhood adversity on women’s health. These findings stress the lasting effect of childhood violence on women’s nutritional status and the urgency of addressing the multiple factors affecting women’s weight through public policy. Strategies should focus on life intervention in infancy and consider the cumulative effects of adversity across the lifespan, with a gender-sensitive approach. Promising actions include targeted support during early childhood, educational initiatives, and comprehensive assistance for families in vulnerable social conditions.

Although IPV is widely recognized as a serious public health concern, our findings did not reveal a statistically significant association between IPV and the outcome. For this purpose, more prospective cohort studies are required to elucidate the complex pathways linking IPV and nutritional health, incorporating more nuanced assessments of violence severity, duration, and timing. Additionally, exploring potential mediators and moderators such as mental health status, social support, and access to healthcare may offer deeper insights into these relationships. We also suggest that future research use more comprehensive measures, including diverse populations, regions, social classes in Brazil, and sex.

## Figures and Tables

**Table 1:** Characteristics of women according to body mass index (BMI). Vitória, Espírito Santo, Brazil, 2022.

Variables	Excess Weight (*n* = 592)	Normal Weight (*n* = 449)	*p*-Value	Extreme BMI (*n* = 268)	*p*-Value
	Mean ± SD	Mean ± SD		Mean ± SD	
Age (years)	48 ± 15	44 ± 17	<0.001	46 ± 15	0.229
Weight (kg)	77.5 ± 11.9	59.2 ± 6.3	<0.001	82.6 ± 17.1	<0.001
Height (cm)	1.61 ± 0.7	1.62 ± 0.7	0.002	1.61 ± 0.7	0.035
Non-white (race/color) (%)	63	55	0.009	64.5%	0.012
Years of education	11.6 ± 4.2	12.8 ± 4	<0.001	11.1 ± 4.1	<0.001
Family income (R$)	4.661 ± 6.339	6.474 ± 8.154	<0.001	3.462 ± 3.465	<0.001
Childhood adversities (%)					
Physical abuse	52	49.2	0.370	51.1	0.623
Emotional abuse	52.5	53.5	0.769	51.1	0.545
Sexual abuse	12.2	10.2	0.334	13.1	0.249
Physical neglect	33.8	30.1	0.204	37.7	0.036
Emotional neglect	52.5	53.5	0.769	51.1	0.545
Abuse of alcohol, illicit drugs, or controlled medication	33.3	24.3	0.002	34.3	0.004
Incarceration of a family member	9	6.5	0.139	10.8	0.038
Depression, mental illness, or suicidal intent	16.6	20.3	0.124	20.2	0.970
Parental separation or divorce	34.3	29.0	0.067	39.9	0.003
Death of parents or guardians	23.7	20.3	0.193	22.4	0.500
Intimate partner violence throughout life (%)					
Psychological	47	42.8	0.178	50.4	0.048
Physical	27	22.9	0.133	32.1	0.007
Sexual	17.4	16	0.560	21.3	0.078

Source: elaborated by the authors; *t*-test and chi-square test.

**Table 2:** Distribution of women with inadequate BMI living in the municipality of Vitória. Vitória, Espírito Santo, Brazil, 2022.

Variables	Excess Weight (BMI ≥ 25 kg/m^2^)				Extreme BMI (BMI < 18.5 or ≥30 kg/m^2^)			
	*n*	P	95%CI	*p*-Value	*n*	P	95%CI	*p*-Value
Age group				<0.001				0.021
18–29 years old	82	43.4	36.5–50.6		42	28.2	21.5–36.0	
30–39 years old	99	50.5	43.5–57.5		59	37.8	30.5–45.7	
40–49 years old	136	63.6	56.9–69.8		60	43.5	35.4–51.9	
50–59 years old	127	67.2	60.2–73.5		52	45.6	36.7–54.9	
60 years or older	148	58.5	52.3–64.4		55	34.4	27.4–42.1	
Ethnicity/skin color				0.009				0.012
White	219	52.0	47.2–56.8		95	32.0	26.9–37.5	
Non-white	373	60.2	56.2–64.0		173	41.2	36.6–46.0	
Years of education				<0.001				<0.001
0–8	135	70.3	44.8–53.5		69	54.8	46.0–63.3	
9–11	208	60.8	55.5–65.9		97	42	35.8–48.5	
12 or more	249	49.1	63.5–76.4		102	28.3	23.9–33.2	
Household income				<0.001				<0.001
1st tertile (poorest)	241	61.2	56.3–65.9		133	46.5	40.8–52.3	
2nd tertile	210	62.9	57.6–67.9		87	41.2	34.8–48.0	
3rd tertile (richest)	141	45.1	39.6–50.6		48	21.8	16.8–27.8	
Adverse childhood experiences								
Physical abuse				0.370				0.623
No	284	55.5	51.1–59.7		131	36.5	31.7–41.6	
Yes	308	58.2	54.0–62.4		137	38.3	33.4–43.4	
Emotional abuse				0.769				0.545
No	281	57.4	52.9–61.7		131	38.5	33.5–43.8	
Yes	311	56.4	52.3–60.5		137	36.3	31.6–41.3	
Sexual abuse				0.334				0.249
No	520	56.3	53.1–59.5		233	36.6	33.0–40.5	
Yes	72	61.0	51.9–69.4		35	43.2	32.8–54.2	
Physical neglect				0.204				0.036
No	392	55.5	51.8–59.2		167	34.7	30.6–39.1	
Yes	200	59.7	54.3–64.8		101	42.8	36.6–49.2	
Emotional neglect				0.769				0.545
No	281	57.4	52.9–61.7		131	38.5	33.5–43.8	
Yes	311	56.4	52.3–60.5		137	36.3	31.6–41.3	
Abuse of alcohol, illicit drugs, or controlled medication				0.002				0.004
No	395	53.7	50.1–57.3		176	34.1	30.1–38.3	
Yes	197	64.4	58.8–69.6		92	45.8	39.0–52.7	
Incarceration of a family member				0.139				0.038
No	539	56.2	53.0–59.3		239	36.3	32.7–40.0	
Yes	53	64.6	53.7–74.3		29	50	37.3–62.7	
Depression, mental illness, or suicidal intent				0.124				0.970
No	494	58.0	54.6–61.3		214	37.4	22.5–41.5	
Yes	98	51.9	44.7–58.9		54	37.2	29.7–45.4	
Parental separation or divorce				0.067				0.003
No	389	54.9	51.2–58.6		161	33.5	29.5–37.9	
Yes	203	61.0	55.6–66.1		107	45.2	38.9–51.6	
Death of parents or guardians				0.193				0.500
No	452	55.8	52.4–59.2		208	36.8	32.9–40.8	
Yes	140	69.6	54.1–66.7		60	39.7	32.2–47.8	
Intimate partner violence throughout life								
Psychological				0.178				0.048
No	314	55.0	50.9–59.0		133	34.1	29.6–39.0	
Yes	278	59.2	54.6–63.5		135	41.3	36.1–46.7	
Physical				0.133				0.007
No	432	55.5	52.0–59.0		182	34.5	30.5–38.6	
Yes	160	60.8	54.8–66.6		86	45.5	38.5–52.7	
Sexual				0.560				0.078
No	489	56.5	53.1–59.7		211	35.9	32.1–39.9	
Yes	103	58.9	51.4–65.9		57	44.2	35.8–52.9	

Source: elaborated by the authors. P: Prevalence. 95%CI: 95% confidence interval.

**Table 3:** Crude and adjusted analysis of the odds ratio of excess weight among women living in the city of Vitória, Espírito Santo, Brazil, 2022 (*n* = 1.041).

Variables	Excess Weight (BMI ≥ 25 kg/m^2^)					
	OR	95%CI	*p*-Value	OR Adj	95%CI	*p*-Value
Age group			<0.001			<0.001
18–29 years old	Ref.			Ref.		
30–39 years old	1.33	0.89–1.99		1.44	0.95–2.16	
40–49 years old	2.28	1.53–3.40		2.44	1.62–3.67	
50–59 years old	2.67	1.76–4.06		2.55	1.66–3.92	
60 years or older	1.84	1.26–2.69		1.68	1.12–2.52	
Ethnicity/skin color			0.009			0.265
White	Ref.			Ref.		
Non-white	1.39	1.08–1.79		1.17	0.89–1.55	
Years of education			<0.001			0.008
0–8	2.45	1.72–3.50		1.88	1.24–2.84	
9–11	1.61	1.22–2.12		1.38	1.01–1.88	
12 or more	Ref.			Ref.		
Household income			<0.001			0.002
1st tertile (poorest)	1.92	1.51–2.83		1.57	1.10–2.25	
2nd tertile	2.07	1.42–2.60		1.82	1.30–2.48	
3rd tertile (richest)	Ref.			Ref.		
Adverse Childhood Experiences						
Physical abuse			0.370			
No	Ref.					
Yes	1.12	0.88–1.43				
Emotional abuse			0.769			
No	Ref.					
Yes	0.96	0.75–1.23				
Sexual abuse			0.334			
No	Ref.					
Yes	1.21	0.82–1.80				
Physical neglect			0.204			
No	Ref.					
Yes	1.19	0.91–1.55				
Emotional neglect			0.769			
No	Ref.					
Yes	0.96	0.75–1.23				
Abuse of alcohol, illicit drugs, or controlled medication			0.002			0.035
No	Ref.			Ref.		
Yes	1.56	1.18–2.04		1.37	1.02–1.83	
Depression, mental illness, or suicidal intent			0.124			0.119
No	Ref.			Ref.		
Yes	0.78	0.57–1.07		0.76	0.54–1.07	
Incarceration of a family member			0.141			0.372
No	Ref.			Ref.		
Yes	1.42	0.89–2.28		1.26	0.76–2.10	
Parental separation or divorce			0.068			0.041
No	Ref.			Ref.		
Yes	1.28	0.98–1.67		1.35	1.01–1.80	
Death of parents or guardians			0.194			0.980
No	Ref.			Ref.		
Yes	1.22	0.90–1.64		1.00	0.73–1.38	
Intimate partner violence throughout life						
Psychological			0.178			0.727
No	Ref.			Ref.		
Yes	1.19	0.93–1.52		1.05	0.80–1.37	
Physical			0.133			0.952
No	Ref.			Ref.		
Yes	1.24	0.94–1.66		1.01	0.70–1.46	
Sexual			0.560			
No	Ref.					
Yes	1.10	0.79–1.53				

Source: elaborated by the authors. OR: odds ratio. OR Adj: odds ratio adjusted. 95%CI: 95% confidence interval. Level 1: age group, ethnicity/skin color, years of education, household income. Level 2: age group, years of education, household income, alcohol abuse, illicit drugs or controlled medications, depression, mental illnesses or suicidal intent, incarcerated relative, separation/divorce of parents, death of parents or guardians. Level 3: age group, years of education, household income, alcohol abuse, illicit drugs or controlled medications, separation/divorce of parents, psychological violence, physical violence.

**Table 4:** Crude and adjusted analysis of the odds ratio of inadequate BMI among women in the city of Vitória, Espírito Santo, Brazil, 2022 (*n* = 717).

Variables	Extreme BMI (BMI < 18.5 or ≥30 kg/m2)					
	OR	95%CI	*p*-Value	OR Adj	95%CI	*p*-Value
Age group			0.023			0.006
18–29 years old	Ref.			Ref.		
30–39 years old	1.55	0.96–2.51		1.74	1.06–2.86	
40–49 years old	1.96	1.20–3.20		2.17	1.30–3.60	
50–59 years old	2.14	1.28–3.57		1.97	1.15–3.37	
60 years or older	1.34	0.82–2.16		1.09	0.64–1.84	
Ethnicity/skin color			0.012			0.867
White	Ref.			Ref.		
Non-white	1.49	1.09–2.03		1.03	0.73–1.46	
Years of schooling			<0.001			0.004
0–8	3.06	1.29–2.59		2.36	1.42–3.92	
9–11	1.83	1.22–2.12		1.42	0.96–2.11	
12 or more	Ref.			Ref.		
Household income			<0.001			<0.001
1st tertile (poorest)	3.11	2.10–4.63		2.29	1.44–3.64	
2nd tertile	2.51	1.65–3.83		2.25	1.44–3.52	
3rd tertile (richest)	Ref.			Ref.		
Adverse Childhood Experiences						
Physical abuse			0.623			
No	Ref.					
Yes	1.08	0.80–1.46				
Emotional abuse			0.545			
No	Ref.					
Yes	0.91	0.67–1.23				
Sexual abuse			0.250			
No	Ref.					
Yes	1.32	0.82–2.10				
Physical neglect			0.036			0.868
No	Ref.			Ref.		
Yes	1.41	1.02–1.94		1.03	0.71–1.50	
Emotional neglect			0.545			
No	Ref.					
Yes	0.91	0.67–1.23				
Abuse of alcohol, illicit drugs, or controlled medication			0.004			0.178
No	Ref.			Ref.		
Yes	1.63	1.17–2.27		1.28	0.90–1.83	
Depression, mental illness, or suicidal intent			0.970			
No	Ref.					
Yes	0.99	0.68–1.45			-	
Incarceration of a family member			0.040			0.333
No	Ref.			Ref.		
Yes	1.76	1.03–3.01		1.34	0.74–2.40	
Parental separation or divorce			0.003			0.009
No	Ref.			Ref.		
Yes	1.63	1.19–2.24		1.59	1.13–2.25	
Death of parents or guardians			0.501			-
No	Ref.					
Yes	1.14	0.79–1.64				
Intimate partner violence throughout life						
Psychological			0.048			0.889
No	Ref.			Ref.		
Yes	1.36	1.00–1.84		1.03	0.69–1.54	
Physical			0.007			0.242
No	Ref.			Ref.		
Yes	1.59	1.13–2.23		1.24	0.86–1.79	
Sexual			0.078			0.913
No	Ref.			Ref.		
Yes	1.42	0.96–2.08		0.97	0.60–1.59	

Source: elaborated by the authors. OR: odds ratio. OR Adj: odds ratio adjusted. 95%CI: 95% confidence interval. Level 1: age group, ethnicity/skin color, years of education, household income. Level 2: age group, years of education, household income, physical neglect, alcohol abuse, illicit drugs or controlled medication, depression, incarcerated relative, separation/divorce of parents. Level 3: age group, years of education, household income, separation/divorce of parents, psychological violence, physical violence, and sexual violence.

## Data Availability

The data presented in this study are available on request from the corresponding author. (The data are not publicly available due to privacy).

## Abbreviations

The following abbreviations are used in this manuscript:

IPV	Intimate Partner Violence
ACEs	Adverse Childhood Experiences
BMI	Body Mass Index
WHO	World Health Organization
